# Meteorological Table

**Published:** 1812-06

**Authors:** 


### METEOROLOGICAL TABLE.

*From April 26, to May 26,* 1812.

| | D. | Therm. | | | Barom. | | Hygrom. *Dry.* | | | *Damp.* | | | Winds. | Atmos. Variation. |
|---|---|---|---|---|---|---|---|---|---|---|---|---|---|---|
| | 27 | 42 | 50 | 48 | $29^6$ | — | 16 | 17 | 15 | | | | E. | C .. R . C . R .. |
| | 28 | 48 | — | 49 | $29^7$ | — | 10 | 12 | — | | | | SE. | C .. R .. — .. — . |
| | 29 | 48 | 53 | 49 | $29^8$ | — | 9 | 12 | 10 | | | | SE.. | R . C ... F ... R ... in N |
| | 30 | 48 | 51 | 48 | $29^8$ | $^9$ | 9 | 1 | — | — | — | 1 | SE. | C ... R .. F . R .. |
| | 1 | 48 | 59 | 50 | 30 | — | — | 15 | 11 | 1 | — | — | N.NE. | C ... F .... C ... |
| | 2 | 49 | 55 | 48 | 30 | $29^8$ | 7 | 13 | 11 | | | | E. | C ... F ... C ... |
| | 3 | 47 | 55 | 49 | $29^8$ | — | 7 | 10 | 9 | | | | NE.. | C ... F .. R .. F .... |
| ◑ | 4 | 44 | 60 | 51 | $29^8$ | — | 9 | 30 | 19 | | | | NE. | F .. — .... — .... |
| | 5 | 48 | 59 | 50 | $29^9$ | 30 | 12 | 15 | — | | | | NE. | F .... — ... C ... F .... |
| | 6 | 48 | 63 | 49 | 30 | — | 11 | 32 | 16 | | | | E. | F .... — .... — .... |
| | 7 | 52 | 59 | 51 | 30 | $29^9$ | 13 | 28 | 18 | | | | E.. | F .. — .... — .... |
| | 8 | 51 | 76 | 61 | $29^9$ | $^8$ | 15 | 40 | 22 | | | | SE.S. | F .... — .... R . in N |
| | 9 | 62 | 69 | 57 | $29^8$ | — | 21 | 39 | 19 | | | | SW... | F ... — ... C ... |
| ● | 10 | 57 | — | 55 | $29^8$ | $^7$ | 15 | 12 | — | — | — | 1 | SW... | F .. C .. R .. — ... in N |
| | 11 | 59 | 65 | 56 | $29^6$ | — | — | 20 | 5 | 9 | — | — | W... | C .... F .... C .... |
| | 12 | 59 | — | 53 | $29^6$ | — | 4 | 14 | 12 | | | | N.NW.. | F .... R .. C ... |
| | 13 | 51 | 57 | 50 | $29^6$ | — | 6 | 16 | 3 | | | | W.. | F ... H .. F ... R ... F .. |
| | 14 | 49 | 59 | 51 | $29^6$ | $^7$ | 1 | 10 | 2 | | | | S.. | C .. F .. — .... |
| | 15 | 51 | 56 | 50 | $29^8$ | $^9$ | 2 | 3 | — | | | | N.SE.. | F .. R .. — .. C ... |
| | 16 | 49 | 65 | 50 | $29^9$ | 30 | 1 | 20 | 7 | | | | NE.. | C ... F .... C ... |
| | 17 | 51 | 54 | 49 | 30 | $29^9$ | | | | 1 | 5 | 6 | NE. | C .... R .. — ... C .... |
| ◐ | 18 | 49 | 58 | 54 | $29^9$ | $^8$ | | | | 5 | 6 | 10 | NE. | R ... F ... — ... |
| | 19 | 55 | 64 | 58 | $29^8$ | $^6$ | | | | 10 | 36 | 26 | E.. | C ... F ... R .... F .. |
| | 20 | 60 | 65 | 58 | $29^7$ | — | | | | 25 | 19 | 26 | W.. | C .. F .. — .... |
| | 21 | 55 | 60 | 53 | $29^8$ | $^7$ | | | | 23 | 20 | 21 | W. | R .. — .. F .. R ... |
| | 22 | 52 | 54 | 47 | 30 | — | | | | 20 | 13 | 15 | N.. | C .. F .. — .... |
| | 23 | 47 | 60 | 47 | $30^2$ | $^3$ | — | 5 | — | 20 | — | 6 | N.E. | F .... — .... — .... |
| | 24 | 53 | 57 | 55 | $30^3$ | $^2$ | | | | 10 | 3 | 6 | W.. | C .. F .. R . F .. |
| | 25 | 56 | 62 | 55 | $30^1$ | 30 | | | | 14 | 27 | — | W. | R . C .. F .. C .. |
| ○ | 26 | 57 | 75 | 65 | $29^9$ | $^8$ | — | 35 | 15 | 27 | — | — | W.. | F ... — .... — ... |

The quantity of rain from the 26th of April to the 26th of May, 2 inches $\frac{65}{100}$; one inch and $\frac{9}{100}$ more than fell from the 26th of March to the 26th of April.

13th. Thunder, with hail and rain. At noon on this day the hail covered the surface of the ground like a fall of snow.

19th. Heavy thunder, incessant lightning, and a deluge of rain in the evening. Preceding these thunder storms, an evident and sudden increase of humidity in the atmosphere, particularly on the 19th, some hours before rain fell.

The diseases of this interval show a gradual decline of those affections which attack the chest. Bronchitis in all its forms, whether acute or chronic, has lessened in frequency. Some cases of Scarlatina have appeared, indicating a disposition to a spread of that disease; and a few solitary instances of Rubeola. This interval may be considered, however, as less productive of disease than the preceding; but we have not data enough yet to pronounce on the modus operandi of atmospherical variations as influencing the state of health or disease.

